# Evidence of High Out of Pocket Spending for HIV Care Leading to Catastrophic Expenditure for Affected Patients in Lao People's Democratic Republic

**DOI:** 10.1371/journal.pone.0136664

**Published:** 2015-09-01

**Authors:** Hubert Barennes, Amphonexay Frichittavong, Marissa Gripenberg, Paulin Koffi

**Affiliations:** 1 Institut de la Francophonie pour la Médecine Tropicale, Vientiane, Lao PDR; 2 Epidemiology Unit, Pasteur Institute, Phnom Penh, Cambodia; 3 Agence Nationale de Recherche sur le VIH et les Hépatites, Phnom Penh, Cambodia; 4 ISPED, Centre INSERM U897-Epidemiologie-Biostatistique, Univ. Bordeaux, Bordeaux, France; Mahidol-Oxford Tropical Medicine Research Unit, THAILAND

## Abstract

**Background:**

The scaling up of antiviral treatment (ART) coverage in the past decade has increased access to care for numerous people living with HIV/AIDS (PLWHA) in low-resource settings. Out-of-pocket payments (OOPs) represent a barrier for healthcare access, adherence and ART effectiveness, and can be economically catastrophic for PLWHA and their family. We evaluated OOPs of PLWHA attending outpatient and inpatient care units and estimated the financial burden for their households in the Lao People's Democratic Republic. We assumed that such OOPs may result in catastrophic health expenses in this context with fragile economical balance and low health insurance coverage.

**Methods:**

We conducted a cross-sectional survey of a randomized sample of routine outpatients and a prospective survey of consecutive new inpatients at two referral hospitals (Setthathirat in the capital city, Savannaket in the province). After obtaining informed consent, PLWHA were interviewed using a standardized 82-item questionnaire including information on socio-economic characteristics, disease history and coping strategies. All OOPs occurring during a routine visit or a hospital stay were recorded. Household capacity-to-pay (overall income minus essential expenses), direct and indirect OOPs, OOPs per outpatient visit and per inpatient stay as well as catastrophic spending (greater than or equal to 40% of the capacity-to-pay) were calculated. A multivariate analysis of factors associated with catastrophic spending was conducted.

**Results:**

A total of 320 PLWHA [280 inpatients and 40 outpatients; 132 (41.2%) defined as poor, and 269 (84.1%) on ART] were enrolled. Monthly median household income, essential expenses and capacity-to-pay were US$147.0 (IQR: 86–242), $126 (IQR: 82–192) and $14 (IQR: 19–80), respectively. At the provincial hospital OOPs were higher during routine visits, but three fold lower during hospitalization than in the central hospital ($21.0 versus $18.5 and $110.8 versus $329.8 respectively (p<0.01). The most notable OOPs were related to transportation and to loss of income. A total of 150 patients (46.8%; 95%CI: 41.3–52.5) were affected by catastrophic health expenses; 36 outpatients (90.0%; 95%CI: 76.3–97.2) and 114 inpatients (40.7%; 95%CI: 34.9–46.7). A total of 141 (44.0%) patients had contracted loans, and 127 (39.6%) had to sell some of their assets. In the multivariate analysis, being of Lao Loum ethnic group (Coef.-1.4; p = 0.04); being poor (Coef. -1.0; p = 0.01) and living more than 100 km away from the hospital (Coef.-1.0; p = 0.002) were positively associated with catastrophic spending. Conversely being in the highest wealth quartile (Coef. 1.6; p<0.001), living alone (Coef. 1.1; p = 0.04), attending the provincial hospital (Coef. 1.0; p = 0.002), and being on ART (Coef.1.2; p = 0.003), were negatively associated with catastrophic spending.

**Conclusion:**

PLWHA’s households face catastrophic OOPs that are not directly attributable to the cost of ART or to follow-up tests, particularly during a hospitalization period. Transportation, distance to healthcare and time spent at the health facility are the major contributors for OOPs and for indirect opportunity costs. Being on ART and attending the provincial hospital were associated with a lower risk of catastrophic spending. Decentralization of care, access to ART and alleviation of OOPs are crucial factors to successfully decrease the household burden of HIV-AIDS expenses.

## Background

The introduction of multidrug antiretroviral therapy (ART), has significantly improved the health status and survival of people living with HIV/AIDS (PLWHA) over the past decade [1,2]. While this has led to reduced hospitalization costs, ART has become the main driver of the total cost of caring for PLWHA. This represents a challenge in low-income countries [3], emerging economies [4,5] as well as in developed countries [6,7]. The price of HIV care can quickly become a considerable economic burden especially in settings where all drugs are not available free of charge, where they remain on charge by the patients or where direct out of pocket payments (OOPs) are required [8]. Evidence shows that HIV treatment and cost of care can be cost effective for emerging countries [5,9–12]. Providing ART drugs free of charge represented an important improvement. However, other indirect costs related to care (such as transportation, loss of income, food etc.) that are not sponsored, and instead are financed by patients through OOPs remain high and represent a financial barrier for health care access in low-resource settings [1,8,12–16] or a reason for decreased treatment adherence [8,15,17,18]. This has an effect on access to treatment, diminishes patients treatment compliance and can further lead to a greater need for complex, expensive second-line antiretroviral drugs [14,15,19,20]. The financial burden of OOPs has disproportionately a higher effect on low-income households [8,21]. Such households have to alter their budgets, reduce consumption of basic commodities and food, which modifies financial and social family dynamics [22]. The economic resources of the family may consequently become consistently exhausted [23].

Catastrophic spending occurs when the ratio between total health expenditure and the difference between income and necessary expenditures (accommodation, food, school, health care and clothing, water, electricity and sanitation) is more than 40%. This increases the risk of impoverishment [24]. Thresholds as low as 10% can also be considered catastrophic. This depends on the economic situation, particularly in low-resource countries. Catastrophic spending affects patients and families in low-resource settings, but also in better-off western countries [25] as well as in emerging Asian economies such as China, Thailand and Vietnam, where universal health coverage schemes exist or are being promoted [15,26–33].

Economic support to patients is becoming an essential component of successful HIV/AIDS programs. It still represents a small proportion of the Global Fund grants to Fight AIDS, Tuberculosis and Malaria [20]. Still, there is limited data available regarding out-of-pocket costs that are incurred by the patient and even less is known about the coping strategies in low-resource countries [14].

In South and South-East Asia approximately 1 028 000 adults and 56 000 child patients are living on ART. In Lao People’s Democratic Republic (Laos, Lao PDR) the proportion of the population living below the official national poverty line dropped from 45% in 1992 to 23.2% in 2012, when the country was classified as a low-middle income country by the World Bank [34,35]. A fragile, limited healthcare insurance system leaves 80% of individuals working in the informal sector without health insurance [34].

We conducted an assessment of the OOPs for health care for PLWHA during hospitalization periods and routine visits in different health facilities in Laos. We assumed that these disbursements for HIV care lead to catastrophic expenditures for PLHIV and their families, in the context of the fragile economic balance of Lao families. We further quantified the extent of catastrophic payments as well as the main coping strategies adopted by patients or their families.

## Methods

### Study site

Laos (6.4 million population) was formerly described as a closed country until it opened up to the market economy in the late 20^th^ century. The per capita income increased from US$ 1,010 per capita in 2011 [36] to US$ 1,460 in 2013 [37–42]. About 80% of the Lao population work in the informal sector, primarily in subsistence agriculture and 44% of the population live below the international poverty line of US$ 1.25 per day.

Public health services were initially mostly composed of very basic free primary care services. User fees for curative public health services were subsequently introduced in the 2000ies. This was followed by the slow reintroduction of user fee exemption for some target population groups, such as the poor. A nationwide, Revolving Drug Fund (RDF) mechanism was established in order to assure a steady supply of essential medicines from the central level all the way down to the village level through cost-recovery. Four health financing schemes were developed: (1) Social Security Organization (SSO) for salaried private employees since 1999; (2) State Authority for Social Security (SASS) for civil servants since 1993; (3) Community-Based Health Insurance (CBHI) for non-poor workers in the informal sector established in 2002; and (4) a Health Equity Fund (HEF) for the poor. However, population coverage by these four main pre-payment schemes is limited to around 19.6% of the population and the overall participation to each scheme is deemed as poor [34]. External funding sources contribute to 40% of the various HEFs. Laos is committed to achieving a form of universal health coverage by 2020. A key step towards universal coverage is the on-going integration of the existing social health protection schemes, which also include a package of free services and drugs for a selection of diseases (Malaria, HIV, Tuberculosis, maternal and child immunization, supplementary nutrients, deworming, etc.).

ART was first introduced in Laos at the Savannaket provincial public hospital (SVK) with the support of “Médecins sans frontières” (MSF) in 2001. It was then gradually extended to public hospitals in the Capital Vientiane, namely to the Setthathirat Hospital (STT) in 2006, and the Mahosot hospital in 2007, in collaboration with MSF, the national HIV/AIDS program, the Center for HIV/AIDS/STI (CHAS) and a French public interest group “Ensemble pour une solidarité thérapeutique hospitalière en réseau” (ESTHER). In 2013, the estimated HIV prevalence among 15 to 49 years old was 0.2%. [43] A total of 2 787 adults and children PLHIV received ART, representing a 58.2% ART coverage.

This study was conducted in SVK hospital in Savannaket province and in STT hospital in Vientiane Capital from March to June 2009. These two hospitals provide ART and treatment for opportunistic infections as well as clinical examinations needed for the follow up treatment (blood cell counts, creatinin, azotemia, transaminase, CD4 count, chest x-ray, and sputum examination for tuberculosis). Viral load testing was not available at the time of the study [44]. Transportation was reimbursed for children and pregnant women in the lowest socioeconomic quintile. At SVK hospital food was provided for inpatients, however not for accompanying persons. The number of PLWHA registered at the SVK was 1,183 and 583 of them received ART the year before the survey (December 2008).

### Study design

We enrolled PLWHA attending an outpatient HIV clinic and HIV inpatients at the infectious disease unit. PLWHA were eligible if they were aged 16 years or above, HIV-positive, and under ART for less than 24 months or awaiting ART. Inpatients were included if it was possible to enroll them at the beginning of their hospitalization period. PLWHA with mental issues or unable to answer questions were excluded.

A list of outpatient attending the HIV clinic was established every day during the survey. From this list of outpatients five patients were selected using a table of random numbers. The number of interviewees was limited to five per day for feasibility reasons.

The procedure differed for inpatients due to the small number of new patient admissions. Newly hospitalized patients in the infectious disease service were subsequently enrolled until a number of 40 inpatients was reached.

We prospectively recorded all OOPs supported by these HIV patients during their outpatient/inpatient period.

All patients were included after informed written consent was obtained.

### Study procedure

An 82-item, pre-tested structured questionnaire was administered to all study participants. The questionnaire addressed the following issues: socioeconomic characteristics, individual and family income and data relevant to expenses during the survey period (including all direct and indirect costs), coping strategies used to face the recurrent expenses of HIV care (loans, sale of assets) and the consequences of the disease on employment.

All expenditures that involved OOPs were recorded including all health-related expenses and indirect expenses. This included transportation costs, number of lost working days and loss of daily income for the patient and accompanying family if any, accommodation, food, consultation fees, purchase of medications, and hospital bills [24]. The characteristics of the disease and patient history since HIV diagnosis were extracted from the patient hospital data base. The inpatients were prospectively asked to complete a full history of their daily expenses related to the current hospitalization period.

### Definitions

Family income was defined as the total revenue of all family members. Income which was not monetary such as farm products (poultry, backyard products, number of rice tons collected for the family) was converted to monetary value. Family income was calculated using the following information: the number of farmers/professionals in the family, the personal report of (monthly or annual) income generated either by farm/shop or production/sales, the number of workers with a salary and the monthly income generated by salaries. The patient income was calculated similarly. From the patient income or the family income we extrapolated the loss of income generated for one day.

Necessary expenditures were defined as the monthly expenses per family for accommodation, electricity, phone, food, school and additional necessary expenses including children care or clothing. Patients were asked to report all these different expenses on a monthly basis.

Necessary expenditures were calculated adding the expenses for the different categories. Capacity to pay was the difference between available income and the necessary expenditures. Total health expenditure was defined as the entire medical or non-medical HIV related expenditures. The medical expenditures included consultations, drugs, and medical exams while the non-medical expenditures concerned transportation and accommodation. This excludes any loss of income related to HIV.

Cost to patients was classified as direct and indirect costs [14]. Direct cost refers to the OOPs medical and non-medical expenditure (medical care, pharmacy, consultations, medical exams, transport etc.).

The indirect costs were defined as the consequences related to the treatment for patients such as loss of work income, loss due to illness, and costs incurred, if any, by the accompanying relative.

Out of pocket payments (OOPs) were defined as all the direct and indirect expenses related to HIV care supported by the patients which were not reimbursed. All financial support/reimbursements received by the patient were recorded and deducted from the OOPs.

Catastrophic costs were defined as OOPs greater than or equal to 40% of the capacity-to-pay [24].

### Data collection

Data collection was done in Lao language by one Lao doctor (AF) attending a master course in epidemiology and trained in infectious diseases.

### Outcomes

The main outcome was the total OOPs. The secondary outcomes were catastrophic expenses, direct and indirect disease costs, costs according to stage of illness, residence, and hospital location, household coping strategies, and impact of the disease on professional activity.

### Sample size

As no published data was available on how to calculate the sample size, the sample size was based on the assumption that the frequency of HIV OOPs account occurred for 50% of the HIV patients attending care in Laos. Using Stata software the sample size was estimated at 257 with 10% precision, alpha = 0.05, power 80%. We added 26 patients (10%) to compensate for non-exploitable files raising the total number to 283 which was rounded down to 280 patients.

### Statistical analysis

Data was entered in EpiData freeware (S1 Dataset). All records were cross-checked with the original data sheets. Analyses were carried out with STATA, Version 8 (Stata Corporation, College Station, TX, USA). Chi squared or Fisher’s exact tests were used to compare categorical variables as appropriate. Data distribution was graphically evaluated using the kemel density estimate and eventually tested with the Skewness and Kurtosis test and the Shapiro-Wilk test for normality. Kruskal-Wallis test was used for non-normally distributed variables. Odds ratio were calculated with exact confidence intervals. We considered p < 0.05 as statistically significant. Means are presented with 95% confidence interval (95%CI) while median are presented with interquartile and range (IQR). Results are presented in US dollars (US$) using the conversion rate of 8500 kip for one dollar of year 2008.

Associations between catastrophic spending and patients’ characteristics were initially measured using bivariate analyses (age, sex, socio-economic conditions, HIV history and status, strategies to cope) and multivariate analyses (S1 Table). Multinomial logistic regression analyses were conducted (S2 Table). Analyses was initially by introducing into the model the variables significantly associated with catastrophic spending in bivariate analysis (those with p-values <0.2, and variables that were assumed to be relevant with catastrophic spending). Variable left in the final model were those with a p-value <0.05.

### Ethics

This study formed part of a master’s course at “Institut Francophone pour la Médecine Tropicale” (IFMT, Vientiane, Laos). All study participants were informed about the study in Lao language and supplied with an information sheet describing the study. Patients were included in the study only after they gave written informed consent. Agreement was recorded on the consent form. Ethical clearance for the study was obtained from the Lao Medical Ethics Committee of the Ministry of Health.

## Results

### Study population

**Fig 1:**
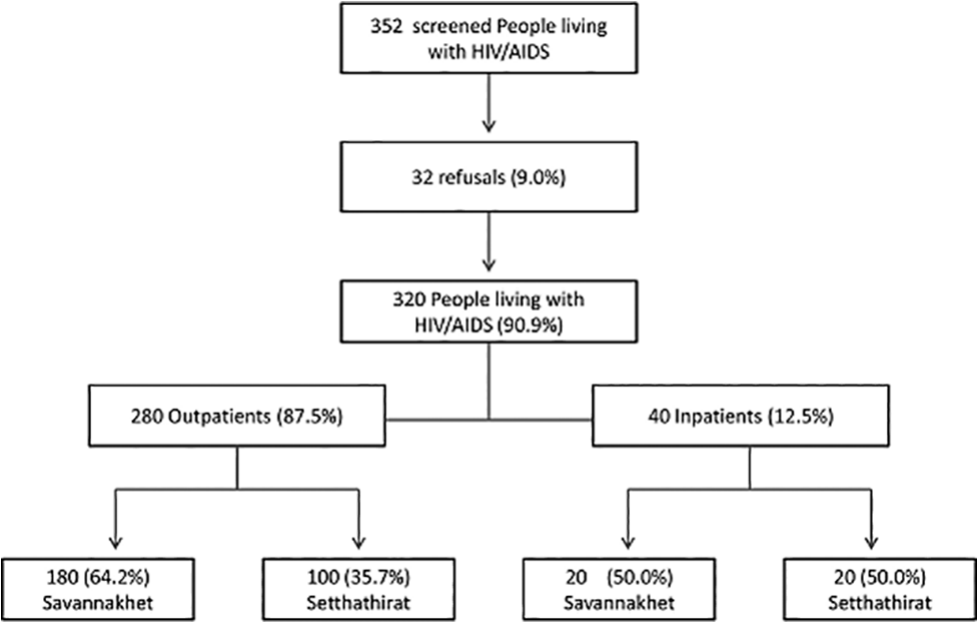
Flow chart of the study population.

A total of 352 patients were screened, 32 (9.1%) refused to participate in the survey leaving 320 patients included for data analysis (Fig 1). This consisted of 280 outpatients and 40 inpatients. The main reason for refusal was lack of time to answer the questions as patients were in a hurry to return home.

The characteristics of the population per hospital are summarized in Table 1. The characteristics of the population per type of care (outpatients/inpatients) are summarized in Table 2. The median age of patients was 33 years (IQR 29–39). The overall sex ratio was 1, but men tended to be more frequently present among the inpatients group (25; 62.5% vs. 15; 37.5%, p = 0.09). Of 320 patients, the majority were from the Lao Loum ethnic group (308; 96.2%), 198 (61.8%) lived in rural areas and 201 (62.8%) were married. The median family size was 5 (IQR: 3–9) without difference according to place of care. A total of 22 patients (6.8%) leave alone and 112 (35.0%) families comprised more than 5 people. Before being diagnosed, 71 patients (22.1%) were farmers, 65 (20.3%) laborers, 59 (18.4%) migrants, 51 (15.9%), shopkeepers 43 (13.4%) employees, 26 (8.1%) unemployed and 5 (1.5%) were students.

**Table 1:** Socio-economics characteristics of patients according to their place of HIV care.

	STT		SVK		
	n = 120	95%CI	n = 200	95%CI	p
**Age** (years)	34.7	33.2–36.1	34.4	33.4–35.5	0.7
**Female** *	62	51.7	98	49	0.6
**Family size**	4.4	4.0–4.8	4.9	4.6–5.3	0.04
Number of children below 15 years	1	0.8–1.2	0.8	0.7–0.9	<0.001
Number of adults unemployed	0.5	0.4–0.6	0.8	0.7–0.9	0.01
**Ethnic group**					
Lao Loum	116	96.7	192	96	0.3
Lao Theung	3	2.5	8	4	
Lao Soung	1	0.8	0	0	
**Urban**	66	55.0	56	28	<0.001
Access to tap water	58	48.3	73	36.5	0.03
**Marital status**					
-Married	77	64.2	125	62.5	0.1
-Single	18	15.0	26	13	
-Divorced	16	13.3	19	9.5	
-Widower	8	6.7	30	15	
**Profession** **					
-None	10	8.3	21	10.5	0.01
-Salesmen	74	61.6	85	42.5	
-Farmer	19	15.8	52	26	
-Migrant	17	14.1	42	21	
Changed profession	82	68.3	112	56	0.02
Stopped working	82	68.3	151	75.50	0.1
**Poor**	27	22.5	105	52.5	<0.001
**Family income** ***	256.1	214.7–297.5	183.6	153.6–213.6	0.004
Essential expenditure	207.0	181.8–232.1	132.5	119.5–145.6	<0.001
Capacity to pay	45.5	17.0–74.0	51.0	26.6–75.5	0.7
**Patient income**	124.0	99.0–149.0	96.2	69.8–122.5	0.1
Loans for HIV	49	40.8	92	46.0	0.3
Loans (US$)	327.4	201.1–453.6	225.7	122.6–328.7	0.2
Sales for HIV	45	37.5	82	41.0	0.5
**Distance to hospital** <100km	95	79.1	107	53.5	<0.001
**Last CD4** (cells/mm3)	205.2	173.4–237.0	187.0	165.7–208.0	0.3
Under ART	100	83.3	169	84.5	0.7
Mean duration on ART (Months)	13.7	11.8–15.5	16.3	14.3–18.3	0.08

Number and percentages

* mean and 95% Confidence interval (95% CI)

** before HIV

*** Income, expenses and capacity to pay per months in US $

**Table 2:** Socio-economic characteristics of people living with HIV/AIDS (outpatients and inpatients) in Lao PDR.

		Outpatients				Inpatients		
	SVK	STT		Total	SVK	STT		Total
	n = 180	n = 100	p	n = 280	n = 20	n = 20	p	n = 40
	(%)	(%)		(%)	(%)	(%)		(%)
**Age (years)**	34.4	34.7	0.7	34.6	34.2	34.3	0.9	34.2
<30	45 (25.0)	29 (29.0)		74 (26.4)	5 (25.0)	4 (20.0)		9 (22.5)
30–39	91 (50.5)	46 (46.0)		137 (48.9)	12 (60.0)	12 (60.0)		24 (60.0)
≥40	44 (24.4)	25 (25.0)	0.7	69 (24.6)	3 (15.0)	4 (20.0)	0.8	7 (17.5)
**Gender**								
Female	89 (49.4)	56 (56.0)		145 (51.8)	9 (45.0)	6 (30.0)		15 (37.5)
Male	91 (50.5)	44 (44.0)	0.2	135 (48.2)	11 (55.0)	14 (70.0)	0.3	25 (62.5)
**Ethnicity**								
Lao Loum	174 (96.6)	96 (96.0)		270 (96.4)	18 (90.0)	20 (100.0)		38 (95.0)
Lao Theung	6 (3.3)	3 (3.0)		9 (3.2)	2 (10.0)	0 (0.0)		2 (5.0)
Lao Soung	0 (0.0)	1 (1.0)	0.4	1 (0.4)	0 (0.0)	0 (0.0)	0.1	0 (0.0)
**Residence**								
Urban	49 (27.2)	56 (56.0)		105 (37.5)	7 (35.0)	10 (50.0)		17 (42.5)
Rural	131 (72.7)	44 (44.0)	<0.001	175 (62.5)	13 (65.0)	10 (50.0)	0.3	23 (57.5)
**Marital status**								
Married	111 (61.6)	65 (65.0)		176 (62.9)	14 (70.0)	13 (65.0)		27 (67.5)
Single	25 (13.8)	12 (12.0)		37 (13.2)	1 (5.0)	6 (30.0)		7 (17.5)
Divorced	17 (9.44)	16 (16.0)		33 (11.8)	2 (10.0)	0 (0.0)		2 (5.0)
Widower	27 (15.0)	7 (7.0)	0.1	34 (12.1)	3 (15.0)	1 (5.0)	0.08	4 (10.0)
**Household income (US$)**	131 (72.7)	74 (74.0)	0.8	205 (73.2)	12 (60.0)	10 (50.0)	0.5	22 (55.0)
Capacity to pay	56 (31.1)	33 (33.0)	0.007	89 (31.8)	4 (20.0)	4 (20.0)	1	8 (20.0)
**Loans for HIV**	83 (94.3)	43 (79.6)	0.7	126 (45.0)	9 (90.0)	6 (100.0)	0.4	15 (37.5)
Loans US$	208.7	340.5	0.1	258.8	374.7	208.9		312.5
	(99.7–317.6)	(201.2–479.7)		173.4–344.2)	(9.0–740.3)	(3.8–421.6)	0.4	(88.1–536.9)
**Sales for HIV**	75 (41.6)	42 (42.0)	0.9	117 (41.8)	7 (35.0)	3 (15.0)	0.1	10 (25.0)
Sales and Loans for HIV	41 (22.7)	24 (24.0)	0.4	65 (23.2)	4 (20.0)	0 (0)	0.9	4 (10.0)
**Main profession before HIV** *								
Farmer	50 (27.7)	16 (16.0)	0.1	66 (23.6)	6 (30.0)	3 (15.0)	0.2	9 (22.5)
Migrant	36 (20.0)	15 (15.0)		51 (18.2)	6 (30.0)	2 (10.0)		8 (20.0)
Unemployed	10 (5.5)	6 (6.0)		16 (5.7)	1 (5.0)	0 (0.0)		1 (2.5)
**Main current profession** *								
Farmer	84 (46.6)	19 (19.0)	<0.001	103 (36.8)	4 (20.0)	4 (20.0)	0.1	8 (20.0)
Migrant	9 (5.0)	4 (4.0)		13 (4.6)	1 (5.0)	1 (5.0)		2 (5.0)
Unemployed	31 (17.2)	19 (19.0)		50 (17.9)	9 (45.0)	4 (20.0)		13 (32.5)
**Changed profession**	104 (57.7)	65 (65.0)	0.2	169 (60.4)	8 (40.0)	17 (85.0)	0.003	25 (32.5)
Stopped working	132 (73.3)	62 (62.0)	0.04	194 (69.3)	19 (95.0)	20 (100.0)	0.3	39 (97.5)
Permanently stopped working	59 (44.7)	20 (32.2)	0.1	79 (28.2)	11 (57.8)	12 (60.0)	0.8	23 (57.5)
**Poor**	96 (53.0)	23 (23.0)	<0.001	119 (42.5)	9 (45.0)	4 (20.0)	0.09	13 (32.5)

Number and percentages, Mean and 95% confidence interval (95%CI), median and interquartile (IQR)

SVK: Savannakhet hospital; STT: Setthathirat hospital; Poor: < 1.25 US $ per day

* We present the results of the frequently most reported professions of the study population

Patients differed in STT and SVK on certain characteristics (family size, number of children below 15 years, number of adults unemployed, access to tap water) (Table 1). SVK patients were more frequently farmers or migrants; they were poorer and living farther away than those in STT. They had been more time on ART. Patients in STT had changed profession more frequently, had a larger family income and spent larger amounts of essential expenditure than compared to those in SVK.

Median household income, essential expenses and capacity-to-pay were US$ 147.0 (IQR: 86–242) per month, $126 (IQR: 82–192) and $14 (IQR: -19–80), respectively. Up to 60% of patients had to change their profession due to their illness and 102 (31.8%) had to stop working permanently, while 131 (40.9%) became temporary workers, after discovering their HIV status. A total of 132 (41.2%) were defined as poor. To face the expenses of the disease, 141 (44.0%) took out loans, and 127 (39.6%) had to sell some of their assets. A total of 45 (14.1%) had to sell gold; 45 (14.1%) cattle; 28 (8.70%) land and 6 (1.9%) had to sell their house. The median amount of loans was US$117 (IQR: 29.4–235) and was higher for outpatients at STT than at SVK (US$340.5; 95%CI: 201–479 versus US$208.8; 173–344). The median distance from home to hospital was 70 km (IQR: 17–200).

Characteristics of patient disease are shown in Table 3. The median length of stay was 1 day (IQR: 1–2) for an external consultation and 16 days (IQR: 4–28.5) for each hospitalization. Hospitalization tended to be of longer duration at STT provincial hospital than at SVK capital hospital.

**Table 3:** WHO Stages, CD4 count and ART status of PLWHA in Lao PDR.

		Outpatients				Inpatients		
	SVK	STT		Total	SVK	STT		Total
	n = 180	n = 100	p	n = 280	n = 20	n = 20	p	n = 40
	(%)	(%)		(%)	(%)	(%)		(%)
**Distance to hospital**								
<100km	102 (56.6)	78 (78.0)	<0.001	180 (64.3)	5 (25.0)	17 (85.0)	<0.001	22 (55.0)
>100km	78 (43.3)	22 (22.0)		100 (35.7)	15 (75.0)	3 (15.0)		18 (45.0)
**Time at hospital (days)** *	1	2	0.9	1	20	15	0.2	16
(IQR)	(1–1)	(1–2)		(1–2)	(12.5–31)	(9–22.5)		(11–28.5)
**WHO stages**								
1–2	31 (17.2)	24 (24.0)	0.1	55 (19.6)	1 (5.0)	0 (0.0)	0.3	1 (2.5)
3–4	149 (82.7)	76 (76.0)		225 (80.3)	19 (95.0)	20 (100.0)		39 (97.5)
**CD4**								
<200 cells/mm3	106 (58.9)	47 (47.0)	0.05	153 (54.6)	16 (80.0)	17 (85.0)	0.6	33 (82.5)
≥200 cells/mm^3^	74 (41.1)	53 (53.0)		127 (45.3)	4 (20.0)	3 (15.0)		7 (17.5)
**On ART**	159 (88.3)	89 (89.0)	0.8	248 (88.5)	10 (50.0)	10 (50.0)	1	20 (50.0)
Time on ART (months)	17.2	15.1		16.4	7.9	6.4	1	7.2
	(15.0–19.3)	(13.2–17.0)	0.9	(14.9–18.0)	(3.2–12.6)	(1.7–11.2)		(3.9–10.4)

Number and percentages, Mean and 95% confidence interval (95%CI)

SVK: Savannakhet hospital; STT: Setthathirat hospital; ART: antiretroviral treatment

* During survey

Almost all patients (268; 83.7%) were on ART, and 264 (82.5%) were at WHO stage 3 or 4. Median CD4 count at initiation of treatment was cells/mm3 (IQR: 19–181) and 90 cells/mm3 (IQR: 25–249) at the time of survey.

Details of household income and of disease related costs are shown in Table 4. A total of 42 (15.0%) outpatients had no revenue and 90 (32.1%) of families earned less than US$100 each month. In the outpatients group, the total cost of each consultation was US$20, of which nearly 50% were direct costs (US$9.6). For each consultation, patients had to pay higher direct cost at SVK (US$10.2) than at STT (US$8.5) (p<0.01). Total OOPs were higher during routine visit at SVK than at STT (US$21.0 versus 18.0, respectively; p = 0.01). Conversely, total OOPs were three fold lower during hospitalization at SVK than at STT (US$110.8 versus US$329.8, respectively, p<0.01). Of 320 patients, 56 (17.5%) received some reimbursement for transport (median: $4.0; IQR: 5.8–9.4). Patients at SVK were more frequently reimbursed for their transportation cost compared to patients at STT in Vientiane (45; 22.5% versus 11; 9.1%, respectively; p = 0.002). Of 280 inpatients, OOPs were classified as catastrophic for 114 (40.7; 95%CI: 34.9–46.7) outpatient’ families.

**Table 4:** Income and out of the pocket spending of PLWHA outpatients and inpatients in Lao PDR.

		Outpatients				Inpatients		
	SVK	STT		Total	SVK	STT		Total
	n = 180	n = 100	p	n = 280	n = 20	n = 20	p	n = 40
	(%)	(%)		(%)	(%)	(%)		(%)
**Family income**	179.9	265.0	<0.01	210.3	217.1	195.5	NS	214.2
	(148.1–211.7)	(216.4–313.6)		(183.2–237.3)	(199.7–314.4)	(141.9–153.1)		(162.8–265.6)
≤100	68 (37.7)	22 (22.0)	<0.01	90 (32.1)	7 (35.0)	4 (20.0)	NS	5.5 (13.7)
100–199	64 (35.5)	33 (33.0)		97 (34.6)	4 (20.0)	6 (30.0)		10 (25.0)
≥200	48 (26.6)	45 (45.0)		93 (33.2)	9 (45.0)	10 (50.0)		9.5 (23.7)
**PLWHA Income**	96.6	135.0	<0.01	110.3	91.7	68.5	NS	80.1
	(67.6–125.6)	(105.8–164.3)		(89.0–131.7)	(47.8–135.6)	(44.7–92.3)		(56.7-103-6)
No revenue	26 (14)	16 (16.0)		42 (15)	6 (30.0)	4 (20.0)		5 (12.5)
≤100$	109 (60.5)	37 (37.0)	<0.01	146 (52.1)	5 (25.0)	11 (55.0)	NS	8 (20.0)
>100$	45 (25.0)	47 (47.0)		92 (32.8)	9 (45.0)	5 (25.0)		7 (17.5)
**Essential expenditure**	129.9	213.3	<0.01	171.6	156.4	175.0		165.7
	(116.3–143.5)	(184.1–242.6)		(145.4–174.0)	(108.3–204.6)	(135.4–204.6)	NS	(135.8–195.6)
**Capacity to pay** *	49.9	51.6	NS	50.7	60.6	20.4	NS	40.5
**Catastrophic costs** **	68 (37.7)	46 (46.0)	NS	114 (40.7)	17 (85.0)	19 (95.0)	NS	36 (90.0)
**Mean direct costs**	10.2 (7.6–12.7)	8.5 (4.9–12.1)	<0.01	9.6 (7.7–11.6)	33.8 (18.1–49.5)	68.5 (44.1–93.0)	<0.01	51.2 (36.2–66.1)
-Medicines	0	0		0	25	0		
-Para clinic test	0.05 (0.0–0.1)	0.2 (0.1–0.4)	<0.01	0.12	3.6 (1.2–6.0)	35.5 (15.7–55.3)	<0.01	23.2
-Transportation cost	9.4 (7.0–16.3)	8.2 (4.6–11.8)	NS	8.8	26.8 (10.5–43.2)	19.9 (6.5–33.2)	NS	
**Mean indirect costs**	10.8 (8.6–12.9)	9.9 (6.6–13.3)	NS	10.3 (8.7–12.3)	77.0 (45.9–108.0)	261.2 (151.7–370.8)	<0.01	169.1 (103.9–225.5)
-Loss of income	4.7 (3.2–6.3)	4.7 (3.1–8.4)	NS	4.3 (3.2–5.5)	53.7 (18.1–110.3)	68.4 (22.7–144.0)	NS	
-Food for illness	3.8 (3.3–4.2)	3.7 (2.1–5.3)	0.04	3.8	78.8 (41.4–166.1)		NS	73.6 (46.1–101.0)
-Housing	0.7 (0.3–1.0)	3.7 (3.0–4.5)	NS	0.4 (0.2–0.6)	-	-		-
**Family loss of income**	2.3 (1.4–3.2)	0.0 (0)	NS	1.5	0.3 (-0.3–1.0)	(39.7–92.1)	NS	0.1 (-0.1–0.5)
-Food for family	0.6 (0.4–0.8)	0.3 (-0.4–1.2)	<0.01	0.7 (0.5–0.9)	16.8 (4.1–29.5)	0 (0–0)	<0.01	23 (0.6–47.2)
-Transport for family	0	0		0	0.3 (-0.3–1.0)	0	NS	
-Housing for family	1.9 (1.1–2.8)	2.6 (0.5–4.7)		2.2	53.5 (28.4–78.7)	194.6 (106.1–283.2)	<0.01	124.0
**Mean costs total**	21.0 (17.1–4.9)	18.5 (12.1–24.9)	0.01	20.1 (16.7–23.4)	110.8 (74.6–7.1)	329.8 (209.7–499.8)	<0.01	220.3 (150.6–289.4)

SVK: Savannakhet hospital; STT: Setthathirat hospital; Number and percentages, Mean and 95% confidence interval (95%CI)

Income, expenses and capacity to pay per months in US $ (US$): 1 = 8500kip

* Difference between family income and essential expenditure

** Number of people where total cost is >40% from their capacity to pay

In the inpatients group, 10 (25.0%) had no revenue and 8 (20.0%) earned less than US$100 per month. The average revenue was US$214.2 (95%CI: 161.4–267.1). The total OOPs for each hospitalization period was US$220.3 (95%CI: 150.8–289.8).

The mean direct costs for hospitalization at SVK (US$33.8) represented half of the total direct costs of US$68.5 at STT (p<0.1). The total average costs at SVK (US$110.8) represented a third of the costs at STT (US$329.8). The OOPs per day was twice as much in the capital STT than in provincial hospital (US$14.3; 95%CI: 11.1–17.5; versus US$7.0 95%CI: 3.9–10.1, respectively p<0.001). Of 40 inpatients, OOPs were considered catastrophic for 36 (90.0%; 95%CI: 76.3–97.2).

Table 5 provides information regarding costs related to CD4 level, WHO stages, and distance to health centers and income. OOPs for consultations were statistically independent from CD4 levels and WHO stages, but dependent on distance and revenue of the patients. For the hospitalized patients, PLWHA in stage 3–4 spent twice as much as those in stage 1–2 (p<0.05). The other aforementioned parameters showed no significant associations.

**Table 5:** Costs according to CD4 level, WHO stages, distance and revenues.

		Outpatients				Inpatients		
		(n = 280)				(n = 40)		
	Direct	Indirect	p	Total	Direct	Indirect	p	Total
	costs	costs		costs	costs	costs		costs
**CD4**								
≤ 200	7.7	9.7	NS	17.5	51.0	189.0	NS	240.0
	(5.8–9.6)	(7.3–12.1)		(13.7–21.2)	(34.0–68.0)	(116.1–261.9)		(157.8–322.2)
> 200	11.8	11.4		23.3	52.1	75.1		127.3
	(7.9–15.8)	(8.7–14.1)		(17.4–29.1)	(11.4–92.8)	(1.7–148.6)		(42.8–211.8)
**WHO**								
stage 1–2	12.6	11.1	NS	23.7	37.7	69.7	<0.01	107.5
	(5.1–20.0)	(7.4–14.1)		(14.3–33.2)	(8.5–66.9)	(17.9–121.5)		(48.6–166.3)
stage 3–4	8.90	10.3		19.2	55.7	201.0		257.9
	(7.0–10.7)	(8.3–12.4)		(15.7–22.7)	(37.6–73.7)	(123.4–280.2)		(169.8–346.0)
**Distance**								
≤100km	3.26	6.1	<0.01	6.19	53.6	212.2	NS	265.9
	(2.2–4.0)	(5.2–7.1)		(5.2–7.1)	(31.3–75.9)	(113.2–311.3)		(154.7–377.0)
>100 km	21.0	18.2		18.2	48.2	116.3		164.6
	(16.1–26.0)	(13.9–22.6)		(13.9–22.6)	(26.7–69.7)	(47.6–185.1)		(86.2–243.0)
**Income**								
≤100$	6.8	7.1	<0.01	14.0	55.1	175.0	NS	230.1
	(5.4–8.2)	(5.9–8.2)		(11.8–16.1)	(34.5–75.6)	(89.5–260.4)		(133.5–326.7)
>100$	15.2	17.4		32.6	43.9	158.1		202.1
	(9.7–20.7)	(12.7–22.0)		(23.8–41.4)	(21.7–66.1)	(64.4–251.9)		(99.9–304.3)

Mean costs and 95% confidence interval (95%CI); US dollars (US$): 1 = 8500kip; CD4: cells/mm^3^

The bivariate analyses of factors associated with catastrophic spending are shown in Table 6. The following factors were associated with catastrophic spending: being from Lao Loum ethnicity, not having access to tap water, living ≥ 100 km away from the hospital, being a migrant or stopped working, being in the 50% quartile of wealthiest or poor, having sold assets for HIV care, not being under ART and more than 6 months on ART.

**Table 6:** Factors associated with catastrophic spending for HIV/AIDS care in Lao PDR (Bivariate analyse).

		Catastrophic spending				
		n	%	Crude OR	95%CI	p
**Sex**	Female	106	66.2	1. (Ref)		
	Male	100	62.5	0.8	0.5–1.3	0.4
**Age (years)**	<20 years	50	60.2	1. (Ref)		
	20–29 years	105	65.2	0.8	0.4–1.4	0.4
	30–39 years	43	68.2	0.7	0.3–1.4	0.3
	40–49 years	8	61.5	0.5	0.09–2.6	0.4
**Ethnicity**	Lao Loum	200	97.0	1.8	0.4–7.0	0.2
	Non Lao Loum	6	50.0	1. (Ref)		
** Living**	Live in family	194	65.2	1. (Ref)		
	Live alone	12	54.5	0.6	0.2–1.7	0.3
	Family size ≤ 6	138	66.3	1. (Ref)		
	Family size > 6 *	68	60.7	0.7	0.4–1.3	0.3
	Not married	71	60.6	1. (Ref)		
	Married	135	65.3	1.2	0.7–2.1	0.2
	Have no child	49	56.9	1. (Ref)		
	Have children	157	67.0	1.5	0.8–2.6	0.09
**Access to tap water**	yes	75	57.2	0.5	0.3–0.9	0.02
	No	131	69.1			
**Environment**	Urban	76	62.3	1. (Ref)		
	Rural	130	65.6	1.1	0.7–1.8	0.5
**Place of care**	Setthathirat	77	64.1	1. (Ref)		
	Savanakhet	129	64.5	1.0	0.6–1.1	0.9
**Distance to hospital**	≤100km	114	56.4	1. (Ref)		
	>100km	92	77.9	2.7	1.5–4.7	<0.001
**Profession before HIV**	Unemployed	16	51.6	1 (Ref)		
	Farmer	44	61.9	1.5	0.5–3.8	0.2
	Migrant	45	76.2	3.0	1.0_8.3	0.01
	Employees	101	63.5	1.6	0.6–3.8	0.1
**Monthly income (quartiles)**	25%	69	83	6.1	2.9–13.7	<0.001
	50%	58	76.3	3.4	1.7–6.7	<0.001
	75%	79	48.4	1. (Ref)		
**Poverty** *	Poor	108	81.8	4.1	2.3–7.3	<0.001
	Not poor	98	52.1			
**Loans for HIV care**	Yes	96	68.4	1.3	0.8–2.1	0.2
	No	110	61.4			
**Sales for HIV care**	Yes	93	73.2	1.9	1.1–3.2	0.007
	No	113	58.5			
**Stopped working**	Yes	159	68.2	1.8	1.0–3.1	0.01
	No	47	54.0			
**WHO stage**	Stage 1	19	52.7	1. (Ref)		
	Stage 2	29	63.0	1.5	0.5–4.0	0.3
	Stage 3	90	66.1	1.7	0.7–3.9	0.1
	Stage 4	68	66.6	1.7	0.7–4.1	0.1
**CD 4 at onset**	≥100 Cells/mm3	120	65.2	1.0	0.6–1.7	0.7
	< 100 Cells/mm3	86	63.2			
**Under ART**	yes	166	61.7	0.4	0.1–0.9	0.02
	no	40	78.4			
**Duration of ART**	<6 months	137	64.3	0.5	0.2–0.9	0.01
	≥ 6months	69	74.1			

Number and percentages; OR and 95% Confidence interval (OR; 95%CI); ART: antiretroviral therapy

*Poor: < 1.25 US $ income per day

After multivariate analysis, 7 factors were independently associated with catastrophic spending (Table 7). The following factors were associated with catastrophic spending: being of Lao Loum ethnic group (Coef.-1.4; p = 0.04); being poor (Coef. -1.0; p = 0.01) and living more than 100 km away from the hospital (Coef.-1.0; p = 0.0020.001). Conversely being in the highest wealth quartile (Coef. 1.6; p<0.001), living alone (Coef.1.1; p = 0.04), attending the provincial hospital (Coef.1; p = 0.002), and being on ART (Coef.1.2; p = 0.003) were associated with lower risk of catastrophic spending.

**Table 7:** Factors associated with catastrophic spending (Multivariate, final model).

	Coef.	Std. Err.	z	P>z
Lao ethnicity	-1.4	0.7	-2.0	0.04
Living below poverty line	-1.0	0.4	-2.6	0.01
> 100 km from HIV centre	-1.6	0.4	-4.8	<0.001
Family income > 75% quartile	1.6	0.4	4.4	<0.001
Live alone	1.1	0.5	2.1	0.04
Care at Savannakhet	1.0	0.3	3.2	0.002
On ART	1.2	0.4	3.0	0.003

Coef. = coefficient, Std. Err. = Standart Error

The groups of 22 people living alone were younger than the others, were mostly businessman or salesmen, (14, 63.6%). Their income was not different from others but they had lower essential expenditures (US$ 108 vs. US$164, p = 0.03).

## Discussion

This survey, set in a low-resource setting, shows the precarity and high level of catastrophic spending frequently faced by PLWHA. Nearly half of outpatients and a third of inpatients were defined as poor. OOPs expenditure reached a catastrophic level for 41% of outpatients and 90% of inpatients compromising an already fragile economic condition. The impact on the work force was also high with up to 60.6% of patients changing profession and 31.8% losing their job. No patients made reference to any form of insurance system and the level of financial support was low and essentially restricted to SVK within an NGO run ward. In the absence of a comprehensive formal social security/insurance mechanism for chronic and long term and repeated treatments, many Lao patients had to resort to their family and traditional social network for alternative support. Such behavior has also been described in other traditional and custom bound societies such as in India [14].

The results show that HIV is associated with the depletion of savings and productive assets and consequent level of households’ debt [14]. The frequent inability of patients to support all HIV- related costs could potentially explain the low coverage of ART in the country and appeals for an appropriate response. We show that PLWHA are faced with high OOPs costs. A previously conducted review described catastrophic health payments in 59 countries, excluding Laos [24]. In this review, Asian countries in transition faced catastrophic spending for health expenditure ranging from 0.8% in Thailand up to 10.4% in Vietnam. Our study offers insight into this issue in Laos. Although not conducted on an entire population level we show high level of catastrophic spending in Laos for HIV/AIDS related to OOPs. Our results prove not surprising as PLWHA face three major components that feed catastrophic spending: poverty, health-service access and use, and the failure of social mechanisms to pool financial risks that do not cover the specific costs linked to HIV/AIDS outside the costs covered by the national HIV/AIS program [24,34].

To improve the situation a few strategies have been developed in Laos including equity funds. These approaches remain largely dependent on external sources and hence are of limited sustainability despite the progressive commitment and funding for health care by the Government of Laos [34]. In addition, these approaches are ill-equipped to provide adequate coverage for people with chronic diseases who require specific long term treatment and repeated health visits.

We evaluated OOPs in the context of two different environments: one urban (STT) and one rural environment (SVK). This was justified by programmatic reasons and the project of scaling up HIV treatment sites to the provincial and districts areas. The assumption behind this comparison was that spending and OOPs differ between the two environments due to the difference of cost of living in Vientiane Capital and in the province. It was important to evaluate and confirm the economic impact for patients in both environments.

We used a conservative definition of catastrophic expense with a higher threshold (40%) than in other studies [24]. This probably underestimates the real economic burden of the HIV disease on households since the majority already live in poverty. In the Lao context, considering a lower threshold for catastrophic expenses may be of interest to protect families from sinking into poverty.

The impact of OOPs was important not only at the patient level but also at the whole family level, particularly for the inpatient group. Despite differences between the province and capital hospitals, each hospitalization can be classified as a catastrophic expense. Over 90% of inpatients faced health expenditures that were of a catastrophic level and likely to lead to severe economic consequences for the family, as opposed to 41% of families of PLWHA attending consultations. Similar studies in other Asian countries such as in neighboring Vietnam, show catastrophic health expenditures for 35.1% of households [1]. Here OOP payments for healthcare are also high and HIV/AIDS patients pay on average US$188 annually for health-care compared to US$165.7 in our study for only one hospitalization period.

Poor people, unlike people in the 75% quartile of wealth, were mostly associated with catastrophic OOPs. This is a frequently occurring trend. A study in India revealed that poor households spent a higher proportion of their income on care and treatment than the better-off households [14]. This study shows that the financial burden of the disease was catastrophic for 59% households below the poverty line and 48% for those above the poverty line. The rate of catastrophic expenses reported in our study is higher than in other countries such as 12.3% in Cote d’Ivoire and 22% for non-insured patients in Senegal [9,13].

Transportation costs were associated with extensive OOP costs for the family. They are a major well-known obstacle for access to treatment and patient adherence reported in many other countries such as Burkina Faso, Cameroon and Malawi [8,16,45,46]. In Vietnam the capacity to pay for ART was influenced by distance from house to clinic, patient’s monthly income as well as insurance status [12]. Supporting patients’ transportation costs is included in the global support package for patients in Cambodia, and may be one of the factors resulting in the success of this program [47,48]. This global package also includes the foremost components such as on site diagnostic tests and treatments, a connected response between all health facilities as well as an extended network of ART sites across the country. Most importantly, economic support to patients is now becoming an important component of various national proposals for the Global Fund to Fight AIDS, Tuberculosis and Malaria grants, if no NGO is able to support this issue [20].

The coping strategies for loss of revenue mostly relied on the sale of assets or contracting family loans. A few families (127, 39.6%) resorted to selling non-monetary valuables (land, cattle, gold). For these families, this suggests that the level of poverty is probably overestimated when using only the daily income of US$1.25 per day, since some families have no revenue but instead non-monetary assets. For other families this suggests that the disease exhausted potential assets and patients had to resort to loans. However it shows the presence of a traditional net of resource that can be used in the case of an emergency. The origin of loans was not investigated and probably need further investigation and we did not know how much of the assets were still left available for the households. These are usually exhausted after the first year of treatment [19]. A similar study in India where no reference to medical insurance was reported, revealed a higher rate of borrowing (67%) and a lower rate of selling assets (8%) than among the Lao patients [14]. In Burkina Faso OOPs for indirect costs of HIV care (including transportation) were described as “social suffering” and entail the difficulty of reaching a quality of life similar to that before becoming ill [23].

The total OOPs for inpatients were three times more expensive in the capital than in the province, while only a small difference was observed for outpatients. The cost of para-clinical tests and medicines for hospitalization was much higher in the capital (US$35.5) compared to the province (US$3.6). The duration of hospitalization was also longer in the capital (24.4 days) compared to the province (14.7 days), which would further add to the cost of treatment. This is partially explained by the support for patients by local NGOs in the province (Savannaket was the first hospital to deliver free antiretroviral drugs in the country, some of the patients received also reimbursement for transportation cost and daily food) and by more sophisticated level of analysis performed in the capital hospital. This supports the recommendation to alleviate indirect OOPs for PLWHA. Similar observations were done in China where the cost of hospitalization was estimated in two provinces [15,34]. Here the cost of treatment had a much higher impact on the annual income of people living in rural areas as opposed to urban dwellers who often receive a higher salary. Results of our study suggest the need for a broader extension of financial support for indirect costs and to not only restrict it to those defined as poor.

The reported level of financial support was lower than expected and variable with patients and sites. This needs to be monitored. In fact, exemptions for the poor and other groups including civil servants and their families as well as monks have been established in Laos [34]. It is left to the discretion of each health facility to decide whether or not to grant a fee exemption. However an evaluation showed a general low implementation level and an unequal distribution between health facilities ranging from 0.3% to 11.9% of total fees [34].

### Perspective

To protect HIV households from catastrophic spending the most straightforward approach suggested by this study is to reduce major out of pocket expenses including transportation and hospitalization costs. Increasing early access to testing and ART would decrease hospitalization costs since here, most patients presented with very low CD4 levels and there is still a need to scale up ART coverage in the country [48,49].

To date, seven main centers and two satellite centers have been gradually established: another one in Vientiane (Mahosot,), 3 in the North (Luang NamTha, Bokeo, Luang Prabang) and another in the South (Pakse)[44]. Due to the size of the country, long distances and the low density of the population and the cost of transportation, these efforts should be scaled up at the primary level in order to bring effective decentralized care closer to people’s homes.

In addition mechanisms to subsidize, through social health protection schemes, indirect costs such as transportation, and compensation for loss of income including of accompanying relatives, such as health equity fund for the poor, are crucial elements to alleviate the burden of the disease faced by PLWHA and their families. To date these mechanisms are not yet in place in Laos (JM Thome, personal communication).

Decreasing patient expenses through the development of social insurance or funding through general taxes is also recommended [24,33]. Total health expenditure per capita is still rather low at US$35 consisting almost of out-of-pocket expenses [34,50,51]. The general government health expenditure has been stagnant up to the year 2011/12 at around 1% of GDP or 4–5% of general government expenditure corresponding to between US$ 6-US$ 10 per capita [34,50,51]. Only about half of the public health expenditure actually came from domestic sources. In the recent years, the Government participation in health spending has started to increase following a commitment to devote 9% of the government expenditure to health. The government health expenditure reached 7% in 2012/13 and is expected to remain stable due to the country’s financial distress [34,52]. This commitment includes the Official Development Assistance (ODA) channelled through the government and progressively the user fees revenues in public health facilities (JM Thome, personal communication). Of interest is the fact that the increase in the budget per capita, which was previously mostly a result of the augmentation of private funding deemed as not sustainable, has now an increased domestic participation that tripled from US$7 in 2011 to US$21 during the financial year 2012/13.

Of concern is the low coverage of the Lao health insurance system and the issue of how to improve its efficiency through better defining the people in need, improved access, and how to avoid impoverished families from facing the huge burden of chronic disease such as HIV but also non communicable diseases such as cancers in the country. A positive aspect is the public and private commitment to improve the situation in the country. This study showed that support for items such as transportation and food, which do not represent a large proportion of health costs, could effectively contribute to help alleviate catastrophic expenses and subsequent impoverishment of families.

### Study limitations

The study has several limitations. Common limitations are related to memory bias and self reporting. Some respondents may have under- or overestimated the costs. To reduce this potential bias, only the costs of the last outpatient visit were recorded and for the inpatient visits, the expenses were prospectively recorded. The study did not investigate in-depth coping strategies among the household (such as who replaced the lost working days income, who in the family sold assets, the origin of assets and of the loans strategies and the level of debts re-imbursements).

## Conclusion

In Laos, the majority of PLWHA households face catastrophic OOPs that are not attributable to the cost of ART or routine follow-up tests. Distance to care, transportation and hospitalizations are the major causes of OOPs. Being on ART and attending the provincial hospital were associated with a lower risk of catastrophic spending. Decentralization of care, access to ART and alleviation of OOPs are crucial factors to successfully decrease the household burden of HIV-AIDS expenses.

## Supporting Information

S1 Dataset Dataset on out of pocket spending for HIV care in lao patients. (XLSX)

S1 Table Variables included in the multinomial regression. (DOCX)

S2 Table Factors associate with catastrophic spending Multivariate analysis. Logistic regression using Odd ratios.
(DOCX)
