# Supplementary material for: Evidence of High Out of Pocket Spending for HIV Care Leading to Catastrophic Expenditure for Affected Patients in Lao People's Democratic Republic
Source: PLoS One. 2015 Sep 1;10(9):e0136664. doi: 10.1371/journal.pone.0136664 (PMC4556637; doi:10.1371/journal.pone.0136664)
Supplement: S1 Table — (DOCX) [file pone.0136664.s002.docx]

Supplementary table 1. Factors associate with catastrophic spending Multivariate analysis

| CATA1YES | Coef, | Std, Err, | P>z | [95% Conf, | Interval] |
| --- | --- | --- | --- | --- | --- |
| SEX | 0,2 | 0,3 | 0,47 | -0,4 | 0,8 |
| AGEGR |  |  |  |  |  |
| <30 | -0,3 | 0,4 | 0,40 | -1,0 | 0,4 |
| 30-39 | -0,7 | 0,5 | 0,11 | -1,6 | 0,2 |
| >40 | -0,2 | 0,8 | 0,77 | -1,7 | 1,3 |
| Rural | 0,4 | 0,4 | 0,34 | -0,4 | 1,1 |
| Savanakhet hospital | 0,4 | 0,4 | 0,34 | -0,4 | 1,1 |
| POOR | -1,0 | 0,4 | 0,023 | -1,8 | -0,1 |
| STOPWORK | 0,0 | 0,3 | 0,99 | -0,7 | 0,7 |
| PROFESSION |  |  |  |  |  |
| Salesmen | -0,2 | 0,5 | 0,65 | -1,2 | 0,8 |
| Farmers | 0,3 | 0,6 | 0,60 | -0,9 | 1,5 |
| Migrants | -1,0 | 0,6 | 0,13 | -2,2 | 0,3 |
| LOAN | 0,2 | 0,3 | 0,45 | -0,4 | 0,8 |
| ETHNIC | -1,3 | 0,8 | 0,10 | -2,8 | 0,2 |
| FAMINCOMEUSD quartile |  |  |  |  |  |
| 25% | 1,6 | 1,2 | 0,20 | -0,8 | 4,0 |
| 50% | 1,7 | 1,2 | 0,16 | -0,7 | 4,1 |
| 75% | 3,3 | 1,3 | 0,011 | 0,8 | 5,8 |
| DISTANCE over 100 km | -1,6 | 0,3 | 0,000 | -2,3 | -0,9 |
| 6 months on ART | 0,5 | 0,3 | 0,16 | -0,2 | 1,2 |
| WHOSTAGEHIV |  |  |  |  |  |
| 2 | -0,7 | 0,6 | 0,22 | -1,8 | 0,4 |
| 3 | -0,5 | 0,5 | 0,34 | -1,4 | 0,5 |
| 4 | -0,6 | 0,5 | 0,27 | -1,5 | 0,4 |
| ONART | 1,1 | 0,4 | 0,01 | 0,3 | 2,0 |
| Access to tap water | 0,2 | 0,3 | 0,57 | -0,5 | 0,8 |
| Live alone | 1,3 | 0,6 | 0,04 | 0,0 | 2,5 |
| SALE of assets | -0,5 | 0,3 | 0,09 | -1,1 | 0,1 |
| _cons | -3,7 | 1,6 | 0,02 | -6,9 | -0,5 |
