# Supplementary material for: Evidence of High Out of Pocket Spending for HIV Care Leading to Catastrophic Expenditure for Affected Patients in Lao People's Democratic Republic
Source: PLoS One. 2015 Sep 1;10(9):e0136664. doi: 10.1371/journal.pone.0136664 (PMC4556637; doi:10.1371/journal.pone.0136664)
Supplement: S2 Table — Logistic regression using Odd ratios. (DOCX) [file pone.0136664.s003.docx]

Supplementary table 2. Factors associate with catastrophic spending. Multivariate analysis (logistic regression using Odd ratios)

|  | OR | Std Err. | Z-value | P |
| --- | --- | --- | --- | --- |
| Lao ethnicity | 3.9 | 2.7 | 2.0 | 0.05 |
| Distance over 100 km from HIV centre | 4.7 | 1.5 | 4.8 | <0.001 |
| Family income, above 75% quartile | 0.2 | 0.1 | -4.5 | <0.001 |
| Patient below poverty line | 2.6 | 1.0 | 2.6 | 0,01 |
| Live alone (n=22) | 0.3 | 0.2 | -2.1 | 0.04 |
| Care at Savannakhet | 0.4 | 0.1 | -3.2 | 0.002 |
| Under ART | 0.3 | 0.1 | -3.0 | 0.003 |
